# 466. Immunogenicity of 2^nd^, 3^rd^, 4^th^ SARS-CoV-2 Vaccines in Renal Patient Populations

**DOI:** 10.1093/ofid/ofad500.536

**Published:** 2023-11-27

**Authors:** Michael Chen-Xu, Daniel Cooper, Rainer Döffinger, Rachel Jones, Rona M Smith

**Affiliations:** University of Cambridge, Cambridge, England, United Kingdom; University of Cambridge, Cambridge, England, United Kingdom; University of Cambridge, Cambridge, England, United Kingdom; University of Cambridge, Cambridge, England, United Kingdom; University of Cambridge, Cambridge, England, United Kingdom

## Abstract

**Background:**

Dialysis, renal transplant (RTX) and autoimmune kidney disease (AKD) patients are at higher risk of COVD-19 and suboptimal humoral responses post primary SARS-CoV-2 vaccination. As such, these groups have been prioritised for further SARS-CoV-2 vaccine doses. Limited data are available on their immunogenicity.

**Methods:**

Patients on dialysis or with a RTX or AKD who received primary SARS-CoV-2 vaccination were prospectively recruited from three UK sites. SARS-CoV-2 IgG spike antibody (anti-S IgG) and nucleocapsid titres were measured with a Luminex assay post 2^nd^, 3^rd^ and 4^th^ SARS-CoV-2 vaccines. Endpoints were median anti-S IgG titres and seroconversion (anti-S IgG titre >1896 MFI).

**Results:**

628 patients (58% male, median age 62 years) comprising 240 dialysis, 194 RTX, and 194 AKD patients were recruited, with seroconversion of 96%, 61% and 70% respectively post two vaccines, and median anti-S IgG titres (IQR) of 30604 (24393–31826), 5829 (455–28296) and 18711 (1233–30226). 400/627 (64%) had anti-S IgG titres post 3^rd^ dose (82% BNT162b2, 7% mRNA-1273, 3% ChAdOx1, 8% unknown; median 191 days post 2^nd^ dose), with 27/58 (47%) of RTX and 17/46 (37%) of AKD patient non-responders seroconverting. Seroconversion among RTX and AKD patients overall improved to 78% (114/146) and 79% (122/155) respectively (p< 0.05), but not in dialysis patients post 3^rd^ dose. 212/400 (53%) had serology post 4^th^ dose (66% BNT162b2, 22% mRNA-1273, 12% unknown; median 104 days post 3^rd^ dose), with 10/22 (46%) and 3/25 (12%) of RTX and AKD patient non-responders seroconverting. Anti-S IgG titre and seroconversion were similar within each group post 4^th^ dose (Table 1, Figure 1).

Predictors of non-seroconversion post 3^rd^ dose in a multivariable model were rituximab use within six months, mycophenolate, prednisolone, chronic kidney disease and AKD (all p< 0.05), and post 4^th^ dose, rituximab alone (p< 0.001).

**Figure:**
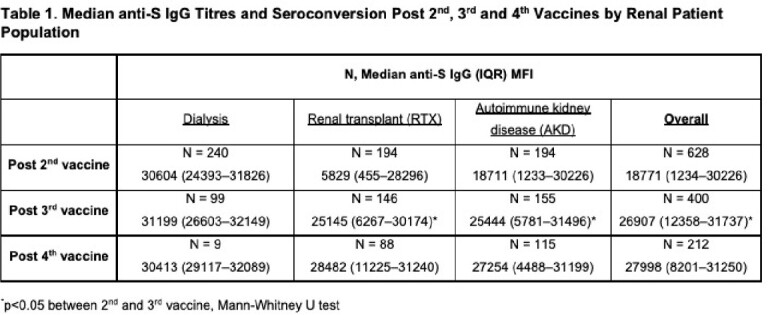

**Figure:**
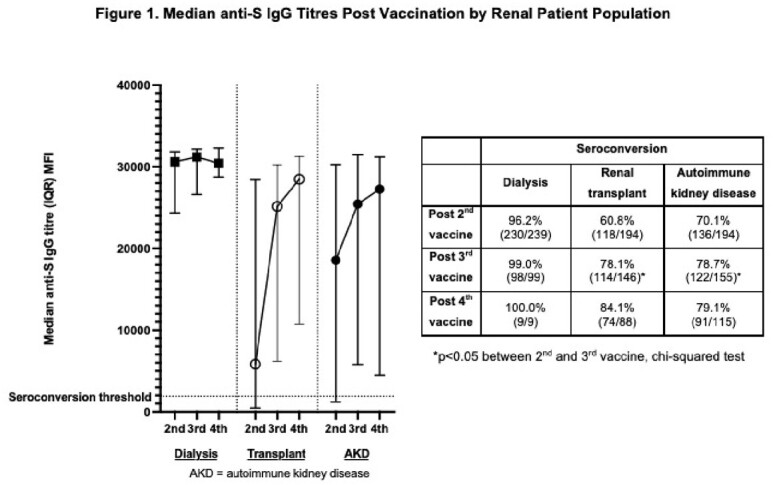

**Figure:**
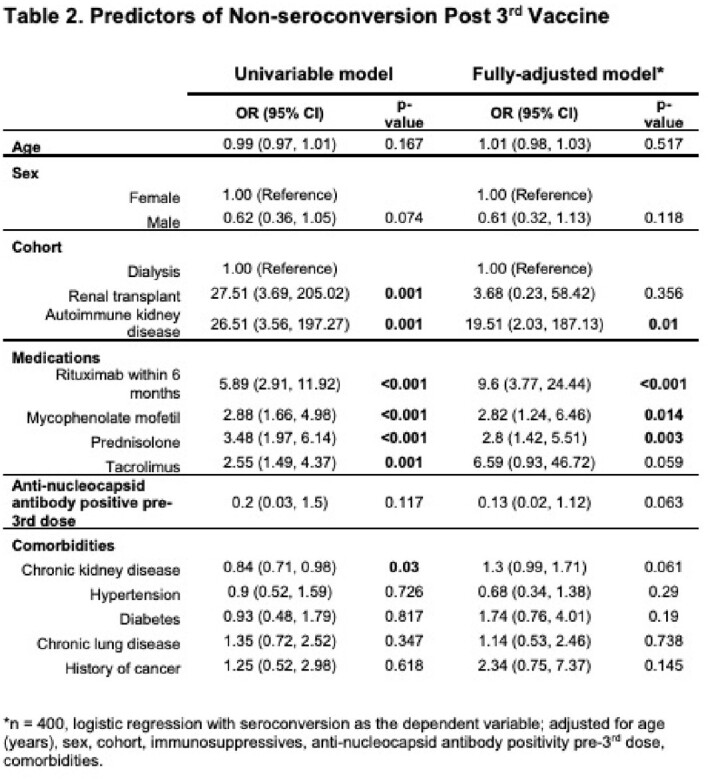

**Conclusion:**

After primary SARS-CoV-2 vaccination, a 3^rd^ but not 4^th^ dose improved anti-S IgG titres and seroconversion in renal patients. Immunosuppressive use, particularly rituximab, was associated with reduced serologic responses (Tables 2, 3). Patients on immunosuppressives should therefore be prioritised for consideration of additional pre-exposure prophylactic agents.

**Figure:**
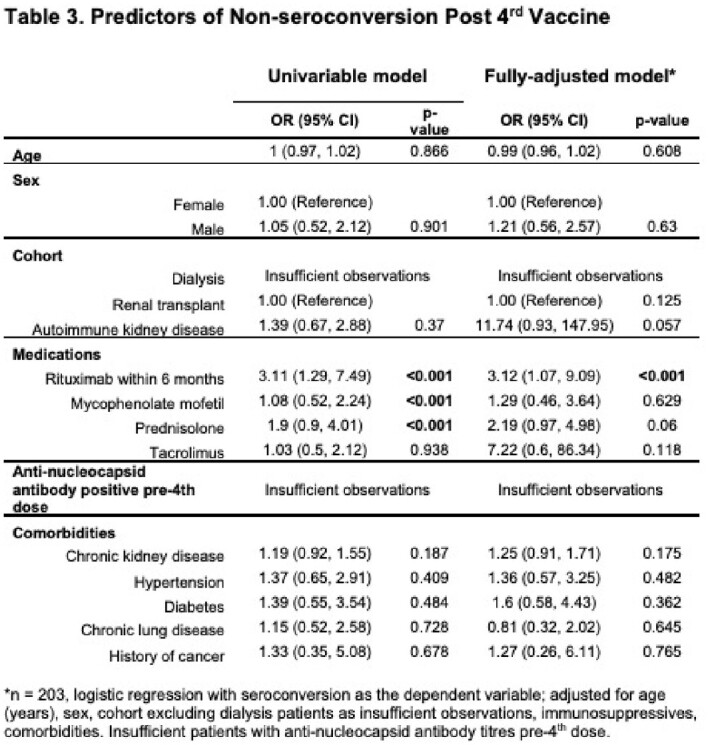

**Disclosures:**

**Michael Chen-Xu, MBChB, MRCP, MPH**, GSK: Grant/Research Support **Rachel Jones, MD**, GSK: Advisor/Consultant|GSK: Grant/Research Support|Roche: Grant/Research Support|Vifor Pharma: Advisor/Consultant **Rona M. Smith, MD MRCP**, GSK: Grant/Research Support|Union Therapeutics: Grant/Research Support

